# Complete Chloroplast Genome Sequence of a Major Invasive Species, Crofton Weed *(Ageratina adenophora)*

**DOI:** 10.1371/journal.pone.0036869

**Published:** 2012-05-11

**Authors:** Xiaojun Nie, Shuzuo Lv, Yingxin Zhang, Xianghong Du, Le Wang, Siddanagouda S. Biradar, Xiufang Tan, Fanghao Wan, Song Weining

**Affiliations:** 1 State Key Laboratory of Crop Stress Biology in Arid Areas, Northwest A&F University, Yangling, Shaanxi, China; 2 College of Agronomy, Northwest A&F University, Yangling, Shaanxi, China; 3 Yangling Branch of China Wheat Improvement Center, Northwest A&F University, Yangling, Shaanxi, China; 4 State Key Laboratory for Biology of Plant Diseases and Insect Pests, Institute of Plant Protection, Chinese Academy of Agricultural Sciences, Beijing, China; Barnard College, Columbia University, United States of America

## Abstract

**Background:**

Crofton weed *(Ageratina adenophora)* is one of the most hazardous invasive plant species, which causes serious economic losses and environmental damages worldwide. However, the sequence resource and genome information of *A. adenophora* are rather limited, making phylogenetic identification and evolutionary studies very difficult. Here, we report the complete sequence of the *A. adenophora* chloroplast (cp) genome based on Illumina sequencing.

**Methodology/Principal Findings:**

The *A*. *adenophora* cp genome is 150, 689 bp in length including a small single-copy (SSC) region of 18, 358 bp and a large single-copy (LSC) region of 84, 815 bp separated by a pair of inverted repeats (IRs) of 23, 755 bp. The genome contains 130 unique genes and 18 duplicated in the IR regions, with the gene content and organization similar to other Asteraceae cp genomes. Comparative analysis identified five DNA regions (*ndhD-ccsA, psbI*-*trnS, ndhF-ycf1, ndhI-ndhG* and *atpA-trnR)* containing parsimony-informative characters higher than 2%, which may be potential informative markers for barcoding and phylogenetic analysis. Repeat structure, codon usage and contraction of the IR were also investigated to reveal the pattern of evolution. Phylogenetic analysis demonstrated a sister relationship between *A*. *adenophora* and *Guizotia abyssinica* and supported a monophyly of the Asterales.

**Conclusion:**

We have assembled and analyzed the chloroplast genome of *A. adenophora* in this study, which was the first sequenced plastome in the Eupatorieae tribe. The complete chloroplast genome information is useful for plant phylogenetic and evolutionary studies within this invasive species and also within the Asteraceae family.

## Introduction

The chloroplasts, considered to be originated from cyanobacteria through endosymbiosis are plant-specific organelles which conduct photosynthesis to provide essential energy for plants and algae [1], [2]. They have their own genetic replication mechanism, transcribe their own genome and carry out maternal inheritance. In higher plants, the cp genome is a circular molecule of double stranded DNA with the size ranging from 120 to 160 kb depending on the species [3]. Generally, the plastid genomes are highly conserved in gene order, gene content, and genome organization in terrestrial plants. The highly conservative nature and slow evolutionary rate of the chloroplast genome demonstrated that it was uniform enough to perform comparative studies across different species but divergent sufficiently to capture evolutionary events, which makes it a suitable and invaluable tool for molecular phylogeny and molecular ecology studies [4].

**Figure 1:**
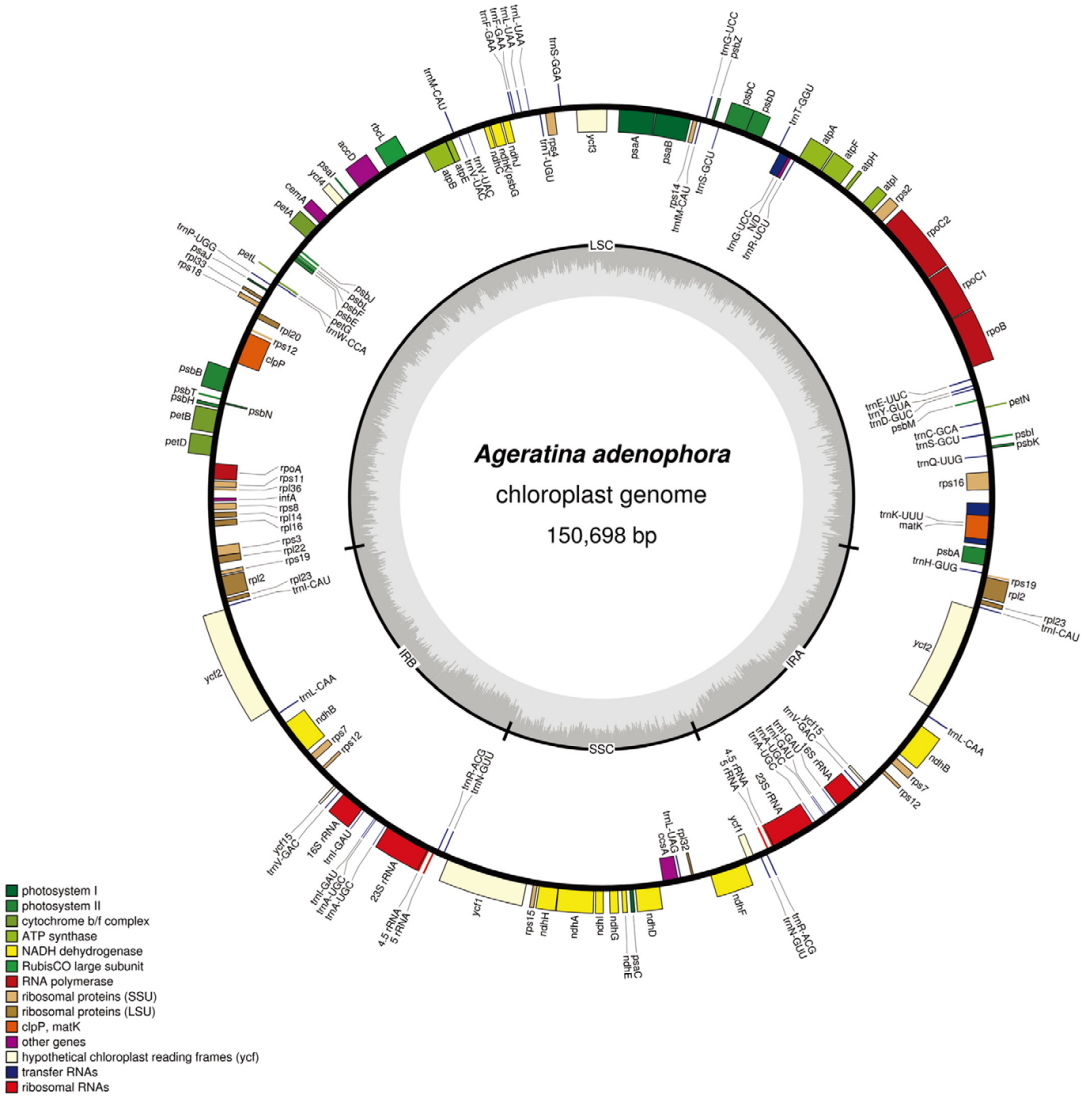
Chloroplast genome map of the *A. adenophora.* Genes lying outside of the outer circle are transcribed clockwise whereas inside are transcribed counterclockwise. Genes belonging to different functional groups are color coded. The innermost darker gray corresponds to GC while the lighter gray corresponds to AT content.

Crofton weed (*A. adenophora*) is perennial herbaceous species, belonging to the Asteraceae family (Eupatorieae tribe). It is native to Central America, ranging from Mexico to Costa Rica, and was introduced to Europe as an ornamental plant in the 19^th^ century and then to Australia and Asia. In the introduced areas, *A. adenophora* is a troublesome species, which inhibits the growth of the local plants and poisons the animals [5]. *A. adenophora* first invaded Yunnan province of China from Myanmar in the 1940's and then rapidly spread to other southern and southwestern provinces of China including Guizhou, Guangxi, Sichuan and Chongqing [6]. Nowadays, it has become the dominant species in local environment, which threatens the native biodiversity and ecosystem, and causes serious economic losses in the invaded areas [7], [8].

During the past two decades, numerous studies using chloroplast DNA sequence data have contributed to our understanding of the evolutionary relationships of angiosperms at species, genera and tribal levels. At the same time, the plastid genome sequence is also the resource of DNA barcodes for plant identification [9] and can be useful in developing informative markers for population studies [10]. The importance of the plastid genome for phylogeny, DNA barcoding, photosynthesis studies and more recently transplastomics [11], has led to sequencing of an increasingly large number of whole chloroplast genomes. Since the first complete chloroplast genome of *Nicotiana tabacum* was published [12], more than 200 complete plastid genomes have been sequenced and analyzed (NCBI, 2011). These chloroplast genomes were mostly sequenced by shotgun sequencing [13] or by conserved primer walking based on the closely related known genome [14]. However, both methods are labor-intensive and time-consuming. With the advent of next-generation sequencing technology, new approaches for chloroplast genome sequencing have been gradually proposed due to their high-throughput, time-saving and of low-cost [15]. For example, the date palm cp genome was sequenced by 454 pyrosequencing [16], duckweed by SOLiD platform [17], and *Jacobaea vulgaris* [18] by Illumina technology.

**Table 1:** Genes present in the *A. adenophora* cp genome.

Gene products		
1	Photosystem I	psaA, B, C, I, J, ycf3^a^, ycf4
2	Photosystem II	psbA, B, C, D, E, F, H, I, J, K, L, M, N, T, Z/lhbA
3	Cytochrome b6/f	petA, B^b^, D^b^, G, L, N
4	ATP synthase	atpA, B, E, F^b^, H, I
5	Rubisco	rbcL
6	NADH oxidoreductase	ndhA^b^, Bb, c, C, D, E, F, G, H, I, J, K
7	Large subunit ribosomal proteins	rpl2b, c, 14, 16, 20, 22, 23^c^, 32, 33, 36
8	Small subunit ribosomal proteins	rps2, 3, 4, 7^c^, 8, 11, 12b, c, d, 14, 15, 16^b^, 18, 19^c^
9	RNAP	rpoA, rpoB, C1^a^, C2
10	Other proteins	accD, ccsA, cemA, clpP^a^, matK, infA
11	Proteins of unknown function	ycf1c, e, ycf2^c^, ycf15^c^
12	Ribosomal RNAs	rrn23^c^, 16^c^, 5^c^, 4.5^c^
13	Transfer RNAs	trnA(UGC)b, c, trnC(GCA), D(GUC), trnE−UUC, F(GAA), G(UCC), H(GUG), I(CAU)^c^, I(GAU)b, c, K(UUU)^b^, L(UAA)^b^, L(UAG), L(CAA)^c^, fM(CAU), M(CAU), N(GUU)^c^, P(UGG), Q(UUG), R(ACG)^c^, R(UCU), S(GCU), S(GGA), T(GGU), T(UGU), V(UAC)^b^, V(GAC)^c^, W(CCA), Y(GUA)

a Gene containing two introns.

b Gene containing a single intron.

c Two gene copies in the IRs.

d Gene divided into two independent transcription units.

e Pseudogene.

**Table 2:** The genes having intron in the *A. adenophora* cp genome and the length of the exons and introns.

Gene	Location	ExonI bp	IntronIbp	ExonII bp	IntronII bp	ExonIII bp
*rps16*	LSC	215	1092	40		
*rpoC1*	LSC	1082	696	646	47	342
*atpF*	LSC	145	852	410		
*ycf3*	LSC	49	898	227	670	125
*clpP*	LSC	228	634	291	809	71
*petB*	LSC	6	774	642		
*rps12* *	LSC	123	-	768		
*petD*	LSC	8	715	475		
*rpl2*	IR	474	457	351		
*ndhB*	IR	756	671	777		
*ndhA*	SSC	552	1097	540		
*trnK*−*UUU*	LSC	35	1559	35		
*trnA*−*UGC*	IR	38	645	31		
*trnL*−*UAA*	LSC	37	437	50		
*trnV*−*UAC*	LSC	37	574	38		
*TrnG*−*UCC*	LSC	47	727	23		
*trnI*−*GAU*	IR	32	739	35		

* rps12 is trans-spliced gene with 5′ end exon located in the LSC region and the duplicated 3′ end exon located in IR regions.

Although five plastid genomes have been sequenced in the Asteraceae family, including *Guizotia abyssinica* [19], *Helianthus annuus* [20], *Parthenium argentatum* [21] (all belonging to the tribe Heliantheae), *Lactuca sativa* [20] (tribe Lactuceae) and *J. vulgaris* [18] (tribe Senecioneae), no plastid genome in the Eupatorieae tribe has been sequenced at present. Here, we reported the complete cp genome sequences of *A. adenophora*, using the Illumina high-throughput sequencing technology. The chloroplast genome sequences will provide helpful genetic tools to conduct population study of *A. adenophora* and help to shed light on the genetic and evolutionary mechanism of the alien species invasion.

**Table 3:** The codon–anticodon recognition pattern and codon usage for *A. adenophora* cp genome.

Amino acid	Codon	No.*	tRNA	Amino acid	Codon	No.	tRNA	Amino acid	Codon	No.	tRNA
Phe	UUU	889	trnF-GAA	Ser	UCU	581	trnS-GGA	Tyr	UAU	736	trnY-GUA
Phe	UUC	510		Ser	UCC	297		Tyr	UAC	174	
Leu	UUA	803	trnL-UAA	Ser	UCA	380		stop	UAA	54	
Leu	UUG	560	trnL-CAA	Ser	UCG	164		stop	UAG	21	
Leu	CUU	567	trnL-UAG	Pro	CCU	411	trnP-UGG	His	CAU	458	trnH-GUG
Leu	CUC	185		Pro	CCC	186		His	CAC	149	
Leu	CUA	370		Pro	CCA	306		Gln	CAA	674	trnQ-UUG
Leu	CUG	157		Pro	CCG	146		Gln	CAG	204	
Ile	AUU	1016	trnI-GAU	Thr	ACU	524	trnT-GGU	Asn	AAU	882	trnN-GUU
Ile	AUC	429		Thr	ACC	233		Asn	AAC	279	
Ile	AUA	642	trnI-CAU	Thr	ACA	382	trnT-UGU	Lys	AAA	888	trnK-UUU
Met	AUG	615	trnM-CAU	Thr	ACG	126		Lys	AAG	314	
			trnfM-CAU								
Val	GUU	495	trnV-GAC	Ala	GCU	643	trnA-UGC	Asp	GAU	806	trnD-GUC
Val	GUC	184		Ala	GCC	222		Asp	GAC	205	
Val	GUA	514	trnV-UAC	Ala	GCA	415		Glu	GAA	916	trnE-UUC
Val	GUG	189		Ala	GCG	151		Glu	GAG	337	
Cys	UGU	209	trnC-GCA	Arg	CGU	345	trnR-ACG	Ser	AGU	389	trnS-GCU
Cys	UGC	73		Arg	CGC	101		Ser	AGC	116	
Stop	UGA	12		Arg	CGA	335		Arg	AGA	451	trnR-UCU
Trp	UGG	439	trnW-CCA	Arg	CGG	113		Arg	AGG	171	
Gly	GGU	586	trnG-UCC	Gly	GGC	196					
Gly	GGA	676		Gly	GGG	293					

* Numerals indicate the frequency of usage of each codon in 24894 codons in 87 potential protein-coding genes.

## Results and Discussion

### Sequencing and Genome assembly

Using the Illumina sequencing technology, we obtained 16, 977, 743 raw reads of 51 bp in length, with 11, 117, 985 unique reads. After filtering for high quality reads, 11, 617, 950 reads with no ambiguous base calls were obtained. Then, we compared two methods to assemble the short-reads sequences. The first one is to assemble the filtered high-quality reads directly with SOAP *de novo* [22] resulting in 12, 161 contigs ranging from 100 to 14, 932 bp. Those contigs were aligned to the *H. annuus* cp genome as the reference genome and 213 contigs had homology with the reference genome with the N50 of 1067 bp. The aligned contigs were ordered according to the reference genome. We obtained a draft sequence of 145, 519 bp in length using this method. The other method is to first capture cp reads from the raw quality-filtered reads (described in Material and Methods). Totally, 1, 815, 199 cp reads were obtained, comprising 90, 759, 950 bp and covering 510.66× *H. annuus* cp genome. Then, 190 contigs ranging from 100 to 8, 810 bp were obtained with the N50 of 2, 221 bp by assembling the captured reads using SOAP. Those contigs were aligned to the *H. annuus* cp genome and ordered consequently. The gaps between them were replaced with the consensus sequences of raw reads mapped to the *H. annuus* cp genome. A draft genome was obtained using this method with the length of 149, 899 bp. To ascertain which method is better, we compared those two genomes with *H. annuus*, *L. sativa* and *G. abyssinica* plastid genomes. Sequence comparison identified that the two sequences assembled by these two methods had 95% sequence identity and the genome assembled by the second method covered some missing regions of the first one. Compared with the *H. annuus* cp genome, the draft genome still contained two gaps. PCR and Sanger sequencing filled the gaps and yielded a complete chloroplast genome of *A. adenophora* with 150, 698 bp in length. To validate the assembly, four junction regions between the IRs and SSC/LSC were confirmed by PCR amplifications and Sanger sequencing. We compared the sequenced results with the assembled genome directly and no mismatch or indel was observed, which supported the accuracy of our assembly. After annotation, this genome sequence has been submitted to GenBank (GenBank ID: JF826503).

**Figure 2:**
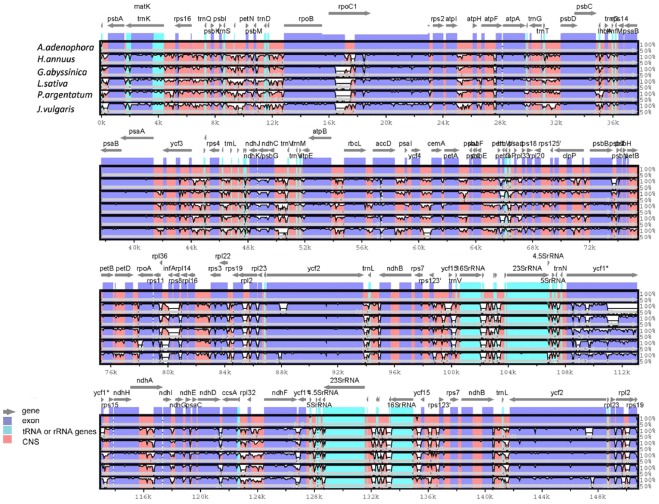
Percent identity plot for comparison of six Asteraceae chloroplast genomes using mVISTA program. Top line shows genes in order (transcriptional direction indicated with arrow). Sequence similarity of aligned regions between *A. adenophora* and other five species is shown as horizontal bars indicating average percent identity between 50–100% (shown on y-axis of graph). The x-axis represents the coordinate in the chloroplast genome. Genome regions are color coded as protein-coding (exon), rRNA, tRNA and conserved non-coding sequences (CNS).

### Genome content and organization

The size of *A. adenophora* cp genome is 150, 698 bp with a typical quadripartite structure, including the LSC of 84, 829 bp and SSC of 18, 359 bp separated by a pair of identical IRs of 23, 755 bp each (Figure 1). The size of *A. adenophora* cp genome is in range with those from other angiosperms. The GC content of *A. adenophora* cp genome is 37.5%, which is consistent with the other reported Asteraceae cp genomes. The GC content of the LSC and SSC region are 35.8% and 30.1%, respectively, whereas that of the IR region is 43.0%.

The *A. adenophora* cp genome contains 80 unique protein-coding genes, seven of which are duplicated in the IR including *rps19, rps7, rpl23, rpl2, ycf2, ndhB* and *ycf15*. Additionally, 28 unique tRNA genes representing all the 20 amino acids are distributed throughout genome (one in the SSC region, twenty in the LSC region and seven in the IR region). Four rRNA genes are also identified in this genome which are completely duplicated in the IR regions. Totally, *A. adenophora* cp genome contains 130 genes (summarized in Table 1). Among them, 14 genes have a single intron (8 protein coding genes and 6 tRNA genes) and 3 genes (*rpoC1, ycf3, clpP*) two introns (all are protein-coding). Out of the 17 genes with introns, 12 are located in the LSC (8 protein-coding and 4 tRNA while 9 have one intron and 3 with two introns), 1 in the SSC (1 protein-coding and has single intron) and 4 in the IR region (2 protein coding and 2 tRNA while all 4 have single intron) (Summarized in Table 2). The *rps12* is a trans-spliced gene with the 5′ end exon located in the LSC region and the duplicated 3′ end exon located in the IR region. The *trnK-UUU* has the largest intron (1, 559 bp) which contains another gene *matK*.

**Table 4:** Promising regions identified for developing phylogenetic markers in Asteraceae family.

Region	Length	Tree	CI	RI	Pars.	Topologies gene
	(bp)	Length		Length	Inf.Char(%)	versus species tree
*accD*	1593	143	0.95	0.82	1.10%	Congruent
*accD*-*psaI*	690	90	0.97	0.90	1.90%	Congruent
*atpA*-*trnR*	121	31	0.95	0.67	2.20%	Congruent
*cemA*	690	50	0.98	0.80	0.70%	Incongruent
*clpP*	2032	196	0.94	0.81	2.60%	Congruent
*ndhA*	2317	129	0.93	0.65	1.30%	Incongruent
*ndhC*-*trnV*	989	341	0.94	0.89	2.30%	Congruent
*ndhD*-*cssA*	242	154	0.93	0.65	4.50%	Congruent
*ndhI*	547	8	0.88	0.50	1.10%	Incongruent
*ndhI*-*ndhG*	350	82	0.99	0.94	3.20%	Congruent
*ndhK*	678	57	1.00	1.00	0.76%	Incongruent
*petB*	1421	76	0.96	0.73	1.45%	Incongruent
*petD*	1197	53	0.98	0.93	1.83%	Congruent
*petN*-*psbM*	468	81	0.96	0.57	1.65%	Congruent
*psbI*-*trnS*	141	26	0.92	0.67	4.04%	Congruent
*psbM*-*trnD*	634	157	0.94	0.62	2.40%	Congruent
*rbcL* *	1458	27	0.89	0.70	1.60%	Congruent
*rps8*-*rps14*	197	88	0.98	0.67	1.80%	Congruent
*rps18*-*rpl20*	245	42	0.98	0.83	1.30%	Incongruent
*trnH*-*psbA* *	415	91	0.93	0.71	3.50%	Congruent
*trnF*-*L* *	347	62	0.95	0.82	3.90%	Congruent
*ycf3*-*trnS*	894	217	0.93	0.62	1.75%	Congruent
*matK* *	1518	85	0.98	0.87	1.84%	Congruent
*rps16* intron*	878	132	0.95	0.74	1.50%	Congruent
Combined regions	20062	2418	0.97	0.83	1.40%	Congruent

* commonly used phylogenetic markers included for comparison.

Sequence analysis indicates 49.56%, 2.32%, and 5.94% of the genome sequences encode proteins, tRNAs, and rRNAs, respectively, whereas the remaining 42.18% are non-coding and filled with introns, intergenic spacers and pseudogenes. Furthermore, the 87 protein-coding genes in this genome represented 74, 682 bp nucleotide coding for 24, 894 codons. On the basis of the sequences of protein-coding genes and tRNA genes within the cp genome, the frequency of codon usage was deduced (Table 3). Among these codons, 2, 642 (10.61%) encode for leucine while 281 (1.12%) encode for cysteine, which are the most and least used amino acids, respectively. The codon usage is biased towards a high representation of A and T at the third codon position, which was similar to the majority of angiosperms cp genomes [23].

### Comparison with other Asteraceae cp genome

From the aspect of genome size, *A. adenophora* chloroplast genome is the second smallest among the six completed Asteraceae cp genomes so far, next to *J. vulgaris* (150, 689 bp). It is around 0.4 kb, 0.77 kb, 2.07 kb and 2.1 kb smaller than *H. annuus, G. abyssinica, L. sativa* and *P. argentatum* genome, respectively. The sequence length variation could be attributed mainly to difference in the length of the LSC and IR regions. It is interesting to find that the *A. adenophora* cp genome contains the largest LSC region among the six cp genomes. But, on the other hand, it has the smallest IR region compared with the other five species.

**Figure 3:**
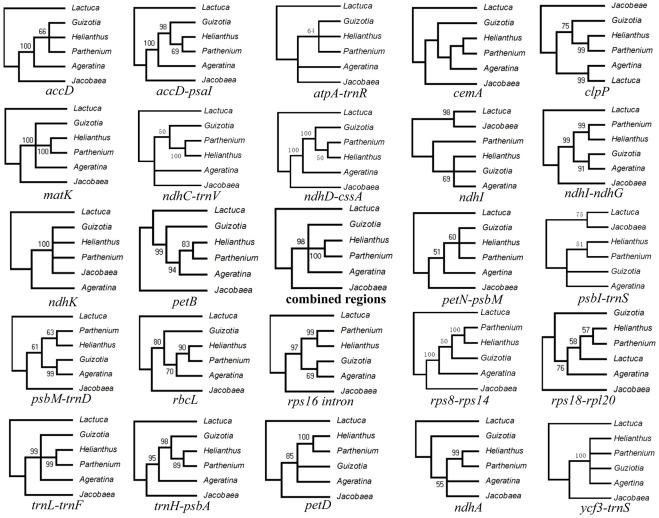
Maximum parsimony (MP) trees of all the selected 24 chloroplast regions of six Asteraceae species . The phylogram of “combined regions” was constructed from the MP analysis using all the 24 regions together.

Compared with other angiosperm species, such as *Arabidopsis* [24] and *Nicotiana* [12], the SSC region is inverted in all of the six Asteraceae cp genomes, which is similar to the *Dioscorea* family [25]. Previous studies demonstrated that a large 23 kb inversion and a smaller 3.4 kb inversion within the large one are observed in the Asteraceae cp genomes. These two inversions were also found in the *A. adenophora* cp genome, indicating that the two inversions maybe present in all Asteraceae species and it may be a key feature of the Asteraceae chloroplast genome. The two inversions were always found together, implying that they occurred together during evolutionary time.

Multiple complete Asteraceae chloroplast genomes available provide an opportunity to compare the sequence variation within the family at the genome-level. The sequence identity of all six Asteraceae chloroplast genome was plotted using the VISTA program with the annotation of *A. adenophora* as reference (Figure 2, Percent identity plot as summarized in Table S2). The whole aligned sequences indicate that the Asteraceae chloroplast genomes are rather conservative, although some divergent regions are found between these genomes. Similar to other angiosperms, the coding region is more conservative than the non-coding counterpart. Of all genes, *rpoC1* gene is the most divergent. *A. adenophora rpoC1* contains two introns, while only one intron is present in each of the other five Asteraceae cp genomes. In addition to *rpoC1*, *ycf1* also shows high sequence divergence. The *ycf1* gene in *A. adenophora* and *P. argentatum* is a pseudogene [21], with high divergence due to various indels. Chloroplast non-coding regions have been proven to work well for phylogenetic studies in angiosperm [26], [27]. Non-coding regions show a higher sequence divergence than coding region among the six chloroplast genomes. In the alignment sequences, a number of regions are found to show high divergence, including *ndhD-ccsA, psbI-trnS, trnH-psbA, ndhF-ycf1* and *ndhI-ndhG*.

**Figure 4:**
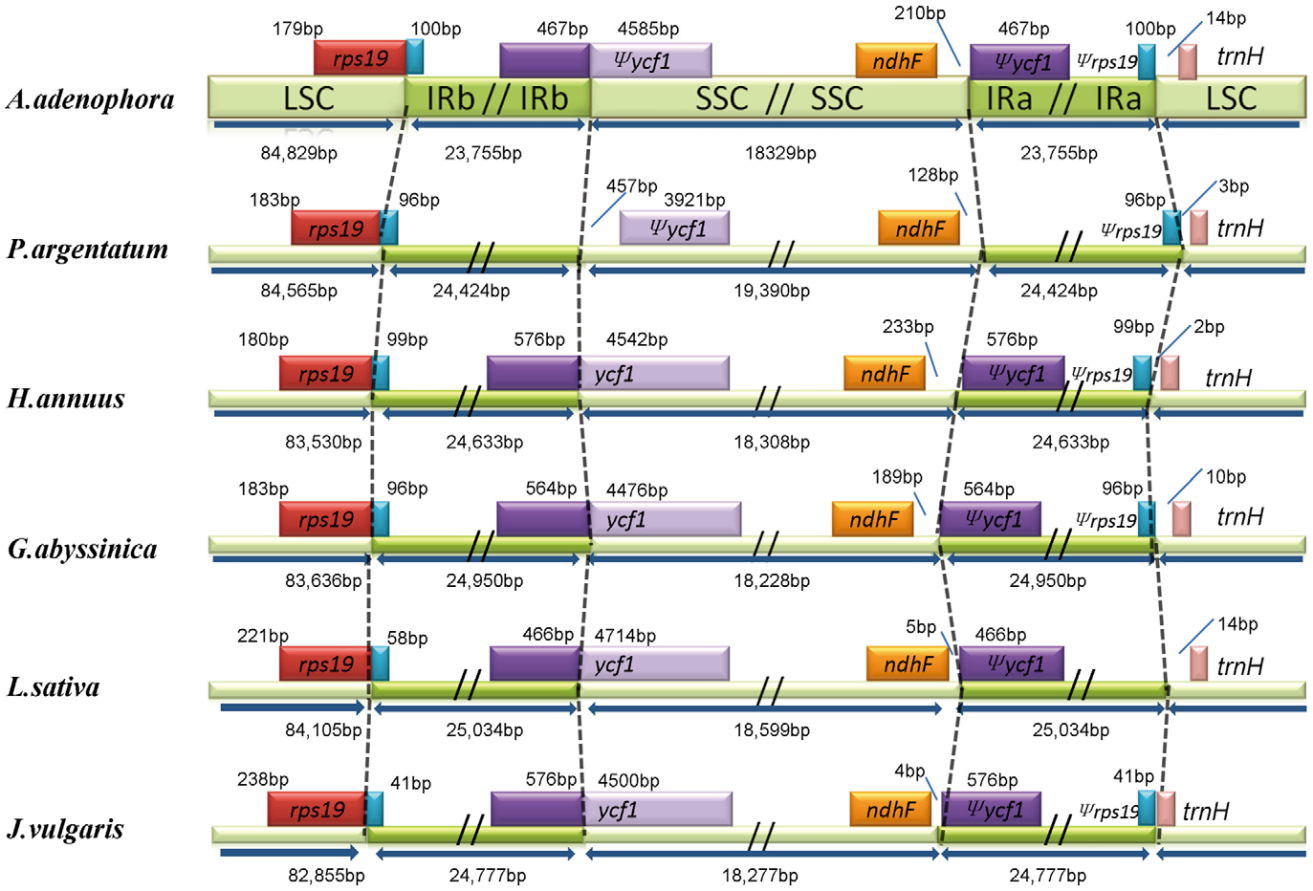
Comparison of the border positions of SSC, LSC, and IR regions among six Asteraceae chloroplast genomes. Selected genes or portions of genes are indicated by the boxes above the genome. The IR regions are extended deep into (576 bp) IRb in the *H. annuus* and *J.* vulgaris chloroplast genomes. Various lengths of *rps19* pseudogene (*ψrps19*) are created at the border of IR/LSC in all of the six chloroplast genomes.

### Identification of molecular markers

Some regions containing sequence divergence were identified during chloroplast genome-wide comparative analysis and they could be suitable for phylogeny study. To examine which regions could be applied to Asteraceae phylogenetic analysis, all of the regions which could be aligned among the six genomes and showed sequence divergence (From Figure 2), alongside the regions frequently used for plant phylogenetic identification (as mentioned in Table 4), were extracted from the 6 Asteraceae chloroplast genomes to perform phylogenetic analysis using the maximum parsimony (MP) method. The result shows that the 6 intergenic regions (*ndhD-ccsA, ndhC-trnV, psbI*-*trnS, ndhI-ndhG*, *atpA-trnR* and *psbM-trnD*) together with commonly used phylogenetic regions (*trnL-trnF* and *trnH-psbA*) contained parsimony-informative characters (Pars.Inf.Char) higher than 2% (Table 4). Among them, the *ndhD-ccsA* region contained the highest Pars.Inf.Char with the value of 4.5%, while that of *trnL-trnF* and *trnH-psbA* were 3.9% and 3.5%, respectively. Compared with the non-coding regions, the protein-coding regions have relatively low Pars.Inf.Char values. Only the *clpP* gene had parsimony-informative characters higher than 2% with the value of 2.6%. The *ndhC-trnV, psbM-trnD* and *clpP* regions have been already identified as divergent regions which contained high phylogenetic information as phylogeny markers in the Asteraceae by previous studies [17]–[19]. The other five regions are newly identified in our current study. Furthermore, many of these regions are not yet used in present molecular phylogenetic studies and may be worthwhile to be adopted in further studies.

**Figure 5:**
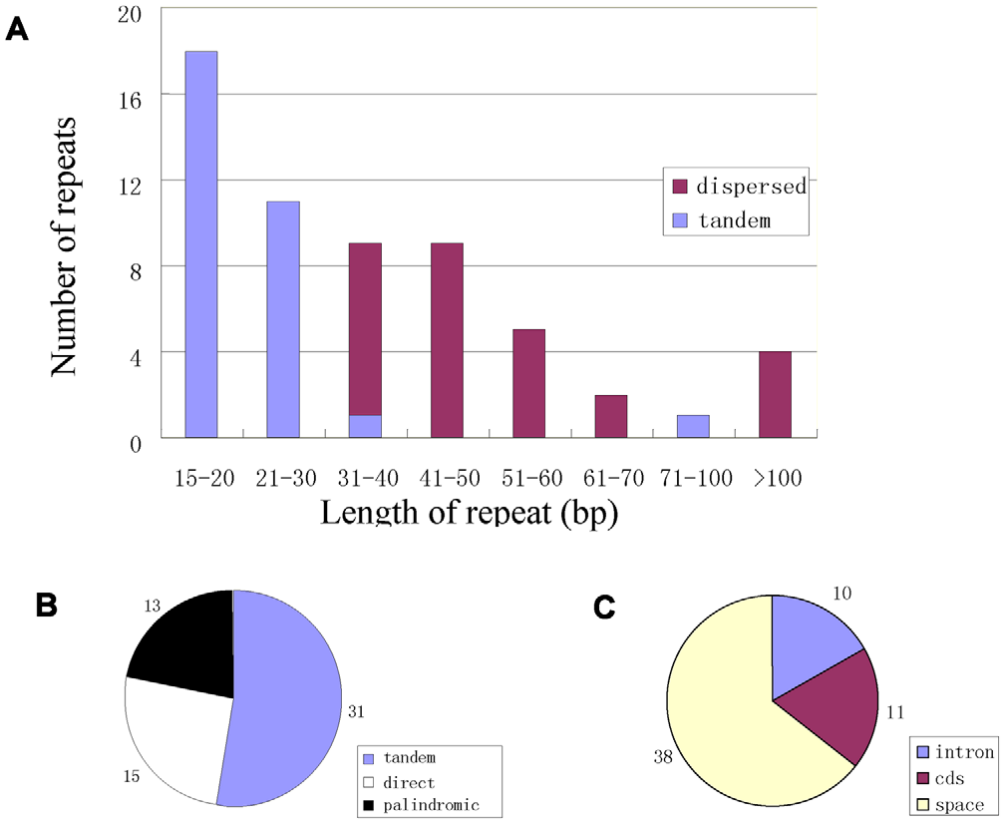
Repeat structure analysis in the *A. adenophora* cp genome. The cutoff value for tandem repeat is 15 bp and 30 bp for dispersed repeat. A. Frequency of repeats by length; B. Repeat type; C. Location distribution of all the repeats.

In general, the phylogenetic trees of the molecular markers should be congruent with that of species because the rates of the sequence evolution are linked to the evolution and life history of species [28]. But when evolution of genes and species did not occur congruently, the gene trees may be incongruent with that of species [29]. To investigate whether our newly identified DNA regions have the congruent trees with the species, the maximum parsimony phylogenetic trees (MPTs) of all the alignable regions with divergence (24 regions in total) were constructed (Figure 3). The results indicate that the genes trees of six regions (*cemA, ndhA, ndhI, ndhK, petB* and *rps18–rpl20*) are incongruent with the combined species trees of Asteraceae family, while all other regions possess the congruent trees.

In this study, some new DNA regions are identified to contain high phylogenetic information and they could be potential molecular marker for phylogenetic analysis. These regions will be particularly helpful for developing universal primers to further reveal the molecular phylogeny of Asteraceae species.

**Figure 6:**
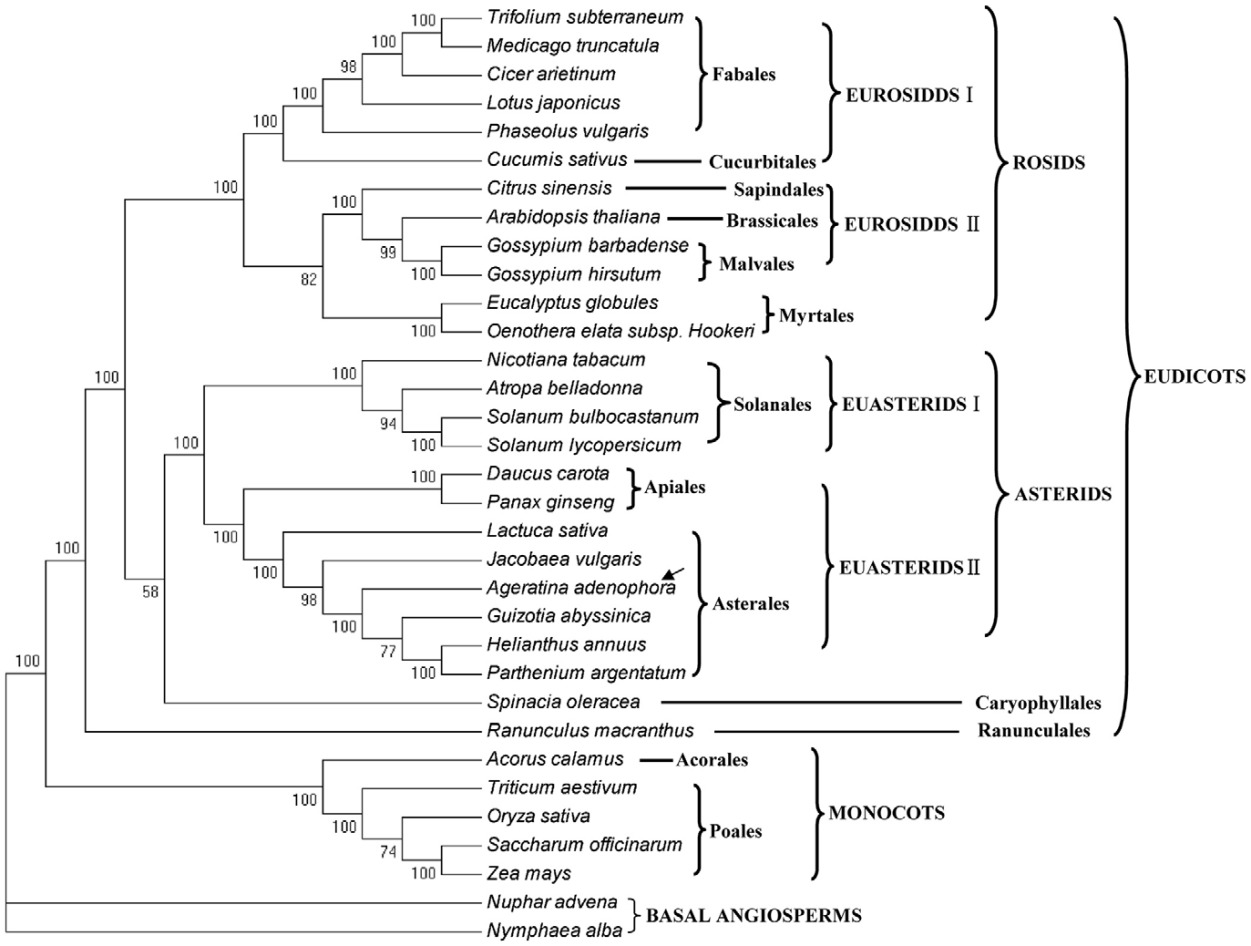
The MP phylogenetic tree is based on 35 protein-coding genes from 33 **plant taxa.** The MP tree has a length of 41, 661 with a consistency index of 0.4644 and a retention index of 0.6821. Numbers above node are bootstrap support values. ML tree has the same topology but is not shown.

### Contraction and expansion of IRs

Generally, the end of the inverted repeats (IRa and IRb) regions differs among various plant species. The contraction or expansion of the IR regions often results in the length variation of the chloroplast genome [30]. The detailed IR-SSC and IR-LSC borders, together with the adjacent genes, were compared across the 6 Asteraceae cp genomes (Figure 4). In all plant species, the border between the IRb and SSC is located in the coding region of *ycf1* gene and results in a pseudogene in the IRa region with the same length as far as the IRb expanded into *ycf1* gene. The IRs of *A. adenophora* expanded 467 bp into the 5′portion of *ycf1* gene, and that of *H. annuus, G. abyssinica, L. sativa and J. vulgaris* expanded 576 bp, 564 bp, 466 bp and 576 bp, respectively. It is very interesting to find that the *ycf1* gene was fully located in the SSC region in *P. argentatum* and 457 bp apart from the IRb/SSC border. In addition to expansion to the *ycf1* gene, the IR region was also expanded to *rps19* gene in all six Asteraceae species. It was expanded 100 bp, 96 bp, 99 bp, 96 bp, 58 bp and 41 bp in *A. adenophora, P. argentatum, H. annuus, G. abyssinica, L. sativa and J. vulgaris,* respectively. The *ndhF* gene was entirely located in the SSC region in all the six species but varied in distance from the IRa/SSC border. The *H. annuus* has 233 bp, the longest intergenic space among these species, whereas *J. vulgaris* has only 4 bp. The position of the *trnH* gene in the cp genome is quite conserved between monocot and dicot species [31]. In general, the *trnH* gene is located in the IR region in the monocots, compared with its location in the LSC region in the dicots. The *trnH* gene of all six Asteraceae cp genomes is located in the LSC region and it is 0−14 bp apart from the IR/LSC border. Overall, although there are minor variations in the contraction or expansion of IR among the Asteraceae family, the IR sequences are not consistent with the total size of plastid genome.

### Repeat structure and sequence analysis

Repeat regions are considered to play an important role in genome recombination and rearrangement [32]. In the current study, we divided the repeats into two categories: tandem and dispersed repeats. After analysis of these repeats in the *A. adenophora* cp genome as described in Material and Methods, 31 tandem repeats were identified with the size not less than 15 bp using the Tandem repeat finder software, of which 18 repeats were 15–20 bp in size, 11 were 21–30 bp, 1 was 32 bp and the rest one was 85 bp (Figure 5A). At the same time, 28 dispersed repeats were also identified, of which 15 were direct repeats and 13 were inverted repeats (palindromic) (Figure 5B). Among the 28 dispersed repeats, 8 were 31–40 bp, 9 were 41–60 bp, 5 were 51–60 bp, 2 were 61–70 bp and the rest were >100 bp in length (Figure 5A). Totally, 59 repeats were identified from the *A. adenophora* cp genome (Table S3). Most of the repeats (64.4%) were distributed within the intergenic spacer regions, together with 16.9% in the introns and 18.7% in the CDS region, respectively (Figure 5C). These repeat motifs will provide very informative source for developing markers for population studies and phylogenetic analysis.

### Phylogenetic analysis

Asteraceae is one of the largest families of angiosperms with approximately 1, 500 genera and 23, 000 species [33]. The plastid sequence is a useful resource for studying the taxonomic status of the Asteraceae in the angiosperm and for analyzing evolutionary relationship within the family. Numerous studies have been conducted to analyze the phylogenetic correlation in Asteraceae, for example Denda *et* *al*. [34] used the *matK* gene to analyze the molecular phylogeny of Asteraceae whereas Panero and Funk [35] combined 10 chloroplast loci from 108 taxa to study the major lineages of Asteraceae. Yet many uncertainties still remains in the molecular phylogeny of Asteraceae and it lacks powerful support and resolution [20]. To obtain reasonable phylogenetic status of the Asteraceae, we performed multiple sequence alignments using protein coding gene from a variety of plant plastomes. Our phylogenetic data set contained 35 coding genes from 33 plant species, including all 6 Asteraceae species. After concatenating alignment, the sequence alignment comprised 35, 114 characters. MP analysis constructed a single tree with a length of 41, 661 with a consistency index of 0.4644 and a retention index of 0.6821. Bootstrap analysis showed that 25 out of 30 nodes have the bootstrap values >95% and 22 of these with the bootstrap values of 100% (Figure 6). Maximum Likelihood (ML) analysis resulted in a single tree with the –lnL of 285544.6056. ML Bootstrap values were high and all the 30 nodes have 100% bootstrap support. MP and ML tree had the same phylogenetic topologies and the phylogenetic tree formed two major clades: monocots and eudicots (Figure 6). Within the eudicots, there were two major clades: rosids and asterids. Then, the rosids clade had two major groups: eurosids I and eurosids II which were sister to the Myrtales group. The phylogenetic position of *Cucumis* was not decided completely in previous studies [36]. In our study, it was belonging to eurosids I because it is sister to the legume taxa, which was comparable to the result of Tangphatsornruang et al. [23]. The asterids clade also had two major groups: euasterids I and euasterids II. All the 6 Asteraceae species were clustered into the Asterales group and placed within the euasterids II, together with the Apiales. This supports a monophyly of the Asterales. Within the Asteraceae family, *A. adenophora* was sister to *G. abyssinica* in the supertribe Helianthodae and was sister to *J. vulgaris* in the subfamily Asteroideae. *L. sativa* was one member of the tribe Lactuceae which was belonging to another subfamily Cichorioideae in the Asteraceae family. The phylogenetic result supports that the tribe Eupatorieae has closer relationship with the tribes Heliantheae and Senecioneae than Lactuceae.

### Conclusion

Using the Illumina high-throughput sequencing technology, we obtained the complete sequence of *A. adenophora* chloroplast genome. It is the first plastid genome sequenced in the *Eupatorieae* tribe and also the sixth in the *Asteraceae* family. Compared with the other Asteraceae chloroplast genomes, this genome has a relative small size, but the organization and gene content is highly similar. Five new regions which contained parsimony-informative characters higher than 2% in addition to 59 repeats were identified, which could be useful for molecular phylogeny and molecular ecology studies within this species and also within Asteraceae family.

## Materials and Methods

### Chloroplast isolation and DNA sequencing

Fresh leaf material was collected from the *A. adenophora* line YN-3 grown at Tengchong County (N 25°52′ 204", E 98°45°220") of Yunnan Province, China after exposure of green plant to dark for two days. Chloroplasts were extracted from the fresh leaves using the protocol developed for sunflower organelle isolation [37]. After DNase treatment, the genomic DNA from chloroplast was isolated using Sarkosyl method [38]. The short-insert sequence library was constructed following the manufacturer's protocol (Illumina, USA). 5 μg of chloroplast DNA was fragmented using dsDNA Fragmentase (NEB, USA) at 37 °C for 30 min, then fragmented DNA was purified using MinElute column (Qiagen, Germany) and eluted in 30 μl elution buffer. Then, T4 DNA polymerase, Klenow polymerase and T4 polynucleotide kinase (Takara, Japan) were added to blunt the DNA fragments at 20°C for 30 min. After purification, an A-tailing was done at 3′ end of the DNA fragments using Klenow fragment and then adaptors (SEQ6+7) were ligated to the end of the DNA fragments using the T4 DNA ligase. Purification of the adaptor-ligated DNA was performed using MinElute column and DNA was eluted in 10 μl ddH2O. DNA fragments ranging between 200–500 bp were recovered from agarose gel using the Gel Extraction kit (Tiangen, China). After purification, PCR was done to amplify the recovered DNA for construction of sequencing library. A single lane of one flow cell was used for sequencing performed on the Illumina GAII according to manufacturer's instructions at Beijing Genomics Institute (BGI) in Shenzhen, China. The sequencing was carried out as single-end run of 51 bp. Further image analysis and base calling were performed using the Illumina Pipeline 1.3.2.

### Genome assembly and annotation

Chloroplast genome was assembled following the method of Zhang *et* *al.* [39] with some modification. The low quality reads of Illumina sequencing were first removed using Perl Script. Then, we compared two methods to assemble the short-read sequence. One is by assembling the quality-filtered read directly into contigs with the minimum length of 100 bp using SOAP de novo [21] with the Kmer = 30, then these contigs were aligned to the *H. annuus* cp genome (used as reference genome) using the BLAST program (http://blast.ncbi.nlm.nih.gov/) and aligned contigs were ordered according to the reference genome. The other method is that we first captured the chloroplast reads from raw quality-filtered reads using BLAST with sunflower (*H. annuus)*, noug (*G. abyssinica*), guayule (*P. argentatum*), lettuce (*L. sativa)* and tobacco (*N. tabacum*) cp genome as query. Then, these captured reads were de novo assembled into contigs with the minimum length of 100 bp using SOAP with Kmer = 30 and then the short contigs were linked into longer one by aligning to *H. annuus* cp genome. Finally, the gaps between the de novo contigs were replaced with consensus sequences of raw reads mapped to the *H. annuus* reference genome. Remaining gaps were filled by PCR and Sanger sequencing using the primers mentioned in Table S1.

The annotation of the cp genome was based on online available program: DOGMA [40], coupled with manual corrections for start and stop codons. The transfer RNA genes were identified by using DOGMA and tRNAscan-SE [41] with default settings. Intron positions were determined following Sugita and Sugiura [42] with those of the *H. annuus* cp genome as reference. The functional classification of cp genes was referred to ChloroplastDB [43]. The circular cp genome map was drawn using the OGDRAW program [44]. To verify the assembly and annotation, the junctions between LSC and IR, SSC and IR were confirmed by PCR and nucleotide sequencing using primers as mentioned in Table S1.

### Comparison with other Asteraceae cp genomes and marker identification

The mVISTA program in Shuffle- LAGAN mode [45] was used to compare the full chloroplast genome of *A. adenophora* with all complete Asteraceae chloroplast genomes (including *H. annuus*, NC007977; *L. sativa*, DQ383816; *P. argentatum*, GU120098; *G. abyssinica*, EU549769 and *J. vulgaris*, HQ234669) using the annotation of the *A. adenophora.* All the regions which could be aligned among six genomes and had sequence divergence were extracted from all six genomes for marker identification. These regions were aligned using ClustalW [46] with further manual adjustment. To get the informative character of these regions, maximum parsimony method was used to construct the phylogenetic tree with Mega4.0 (gap opening penalty: 15; gap extension penalty: 6.66; DNA weight matrix: IUB; transition weight: 0.5; negative matrix: off; and delay divergent cutoff: 30%) [47]. Bootstrap consensus tree was inferred from 1, 000 replicates [48]. Branches corresponding to partitions reproduced in <50% bootstrap replicates are collapsed. The values of replicate trees in which the associated taxa clustered together in the bootstrap test (1, 000 replicates) are shown next to the branches. Parsimony-informative characters, consistency index (CI) and retention index (RI) values were also calculated.

### Repeat structure and sequence analysis

Repeat structure of *A. adenophora* chloroplast genome was analyzed following the Zhang *et* *al.*'s method [39] with some modification. Tandem repeats were analyzed using Tandem repeat Finder program [49] with the parameters setting as 2, 7 and 7 for match, mismatch and indel respectively. The minimum alignment score and maximum period size were set as 50 and 500 respectively. REPuter [50] was used to identify and locate disperse repeat including the direct (forward) and inverted (palindrome) repeats with the setting that identity of the repeat was no less than 90% (hamming distance equal to 3) and the size of repeat was more than 30 bp, respectively. After program analysis, tandem repeats with less than 15 bp in length and the redundant results of REPuter were manually removed.

### Phylogenetic analysis

A set of 35 protein-coding genes atpA, atpB, matK, petA, petB, petD, petG, petN, psaA, psaB, psbA, psbB, psbC, psbD, psbE, psbF, psbH, psbI, psbJ, psbK, psbN, psbT, rpoB, rpoC1, rpoC2, rps8, rps11, rps14, ycf3, ndhA, ndhD, ndhH, ndhF, rpoA and rbcL from 33 cp genomes representing all lineages of angiosperms were used for phylogenetic analysis. These 35 genes are commonly present in all 33 publicly available cp genomes (Listed in Table S4) in the GenBank database. Sequences were aligned using ClustalW and alignment was edited manually. MP analysis was performed with PAUP*4.10 [51] (http://paup.scs.fsu.edu/) using heuristic search, random addition with 1, 000 replicates and tree bisection-reconnection (TBR) branch swapping with the MulTrees option. Non-parametric bootstrap analysis was conducted under 1, 000 replicates with TBR branch swapping. Maximum likelihood (ML) analysis was carried out in PhyML v3.0 [52]. The general time reversible (GTR) model [53] of nucleotide substitution was selected for ML analysis, taking in account the gamma distribution of rate heterogeneity with four discrete categories [54]. The robustness of tree nodes was assessed using 1000 non-parametric bootstrap pseudo-replicates. Nuphar and Nymphaea were used as the out-group.

## Supporting Information

Table S1 **Primers used for gap filling and assembly validation.**
(DOC)

Table S2 **Comparison with the homologues between the A. adenophora cp genome** **and** *Helianthus annuus (Ha), Lactuca sativa (Ls), Guitozia abyssinica (Ga), Parthenium argentatum (Pa)* **and** *Jacobaea vulgaris (Jv)* **by the percent identity of coding and non-coding regions.**
(DOC)

Table S3 **Repeat sequences in the** ***A*** **.** ***adenophora*** **chloroplast genomes.**
(DOC)

Table S4 **The GenBank accession numbers of all the 33 cp genomes used for phylogenetic analysis.**
(DOC)
