# Supplementary material for: Complete Chloroplast Genome Sequence of a Major Invasive Species, Crofton Weed (Ageratina adenophora)
Source: PLoS One. 2012 May 11;7(5):e36869. doi: 10.1371/journal.pone.0036869 (PMC3350484; doi:10.1371/journal.pone.0036869)
Supplement: Table S1 — Primers used for gap filling and assembly validation. (DOC) [file pone.0036869.s001.doc]

**Table S1.** Primers used for gap filling and assembly validation.

| **Primer^*^** | **Sequence (5’>3’)** | **Amplicon size** |
| --- | --- | --- |
| 1 | F: AGTTACTAATTATGATCTGGCATGT  R: ATAGCAATGAGATTCCCCAA | 579 |
| 2 | F: TGGGGAGAAATGACAAGA  R: TTTATCTTATTATTAATCACGGA | 1089 |
| 3(IR1/LSC) | F: TGT TGA CCT AAA GCG TAT  R: TGA ACC CTG TAG ACC ATC | 463 |
| 4(LSC/IR2) | F AAG CGT TGG CTA GGT AAG  R: TAG CCA AGT GGA TCA AGG | 896 |
| 5(IR2/SSC) | F CCA GGG CTA TGG AAC AAA G  R: AAA CCA CAA CGA CCG AAT | 652 |
| 6(SSC/IR1) | F: CTC GCT AAC ATT GAA CTT GG  R: ACC TCC CGT TCT TCA TAC TT | 660 |

^*^Primer pairs 1 and 2 were used to fill the gaps of the assembled genome; Primer pairs 3-6 were used to verify the junctions between the IRs and SSC/LSC regions.
