# Supplementary material for: Complete Chloroplast Genome Sequence of a Major Invasive Species, Crofton Weed (Ageratina adenophora)
Source: PLoS One. 2012 May 11;7(5):e36869. doi: 10.1371/journal.pone.0036869 (PMC3350484; doi:10.1371/journal.pone.0036869)
Supplement: Table S2 — Comparison with the homologues between the A. adenophora cp genome and Helianthus annuus (Ha), Lactuca sativa (Ls), Guitozia abyssinica (Ga), Parthenium argentatum (Pa) and Jacobaea vulgaris (Jv) by the percent identity of coding and non-coding regions. (DOC) [file pone.0036869.s002.doc]

**Table S2. Comparison with the homologues between the *A. adenophora* cp genome and *Helianthus annuus* (*Ha*), *Lactuca sativa* (*Ls*), *Guitozia abyssinica* (*Ga*), *Parthenium argentatum(Pa)* and *Jacobaea vulgaris*(Jv*)* by the percent identify of coding and non-coding regions.**

| Name | Type | Start | End | Length  (bp) | %Identify | | | | |
| --- | --- | --- | --- | --- | --- | --- | --- | --- | --- |
|  |  |  |  |  | *Ha* | *LS* | *Ga* | *Pa* | *Jv* |
| ycf1 | gene | 108103 | 113154 | 5051 | 84.30 | 85.20 | 96.00 | 96.00 | 91.60 |
| ycf2 | gene | 86995 | 93741 | 6813 | 91.70 | 94.80 | 92.70 | 96.10 | 90.60 |
| accD | gene | 56737 | 58329 | 1593 | 91.20 | 96.20 | 93.60 | 97.85 | 95.30 |
| ndhK | gene | 48696 | 49373 | 678 | 92.30 | 94.30 | 96.60 | 94.80 | 90.00 |
| rpl14 | gene | 80993 | 81370 | 378 | 92.20 | 93.20 | 92.70 | 92.20 | 89.90 |
| rps3 | gene | 83074 | 83730 | 1124 | 94.20 | 94.20 | 94.70 | 94.00 | 93.10 |
| rpl22 | gene | 83715 | 84188 | 474 | 95.00 | 95.20 | 96.50 | 96.90 | 91.80 |
| rpl36 | gene | 79506 | 79622 | 117 | 95.40 | 97.40 | 96.50 | 93.20 | 93.20 |
| petG | gene | 65901 | 66014 | 114 | 96.50 | 94.60 | 95.60 | 94.30 | 96.50 |
| matK | gene | 2096 | 3613 | 1518 | 96.60 | 95.70 | 97.60 | 97.00 | 94.60 |
| petL | gene | 65598 | 65693 | 96 | 96.90 | 93.80 | 97.90 | 97.90 | 97.90 |
| rps19 | gene | 84637 | 84914 | 278 | 97.10 | 98.90 | 98.90 | 97.80 | 97.80 |
| ndhF | gene | 124495 | 126720 | 2211 | 97.20 | 97.30 | 97.80 | 97.60 | 95.50 |
| psbH | gene | 74365 | 74586 | 222 | 97.30 | 97.30 | 97.70 | 98.60 | 95.90 |
| rps15 | gene | 113548 | 113826 | 279 | 97.80 | 96.40 | 97.10 | 97.80 | 95.30 |
| petA | gene | 61832 | 62793 | 962 | 97.90 | 98.20 | 98.40 | 98.20 | 97.30 |
| rpl20 | gene | 68203 | 68583 | 381 | 97.90 | 97.10 | 98.20 | 98.20 | 97.10 |
| rbcL | gene | 54798 | 56231 | 1436 | 98.10 | 97.80 | 98.70 | 98.50 | 96.70 |
| ndhD | gene | 119810 | 121310 | 1501 | 98.10 | 97.50 | 98.50 | 98.50 | 97.10 |
| clpP | gene | 69585 | 71616 | 591 | 98.10 | 98.00 | 98.20 | 98.20 | 97.30 |
| rpoC2 | gene | 18916 | 23073 | 4158 | 98.20 | 96.90 | 98.30 | 98.10 | 96.30 |
| psaI | gene | 59020 | 59130 | 111 | 98.20 | 97.30 | 98.20 | 98.20 | 98.20 |
| psbT | gene | 73956 | 74069 | 114 | 98.20 | 96.50 | 100.00 | 98.20 | 98.20 |
| rps11 | gene | 79038 | 79424 | 387 | 98.20 | 98.20 | 98.80 | 99.00 | 98.80 |
| ccsA | gene | 121553 | 122521 | 969 | 98.20 | 95.30 | 98.80 | 98.60 | 94.60 |
| rps16 | gene | 5191 | 5404 | 254 | 98.40 | 99.00 | 99.30 | 98.40 | 98.20 |
| rpoC1 | gene | 16046 | 18856 | 2070 | 98.40 | 98.00 | 98.23 | 98.33 | 97.70 |
| ndhJ | gene | 48122 | 48598 | 477 | 98.50 | 97.50 | 99.40 | 98.30 | 97.70 |
| rps8 | gene | 80435 | 80839 | 405 | 98.50 | 97.30 | 99.30 | 98.80 | 97.50 |
| rpoA | gene | 77950 | 78957 | 1008 | 98.60 | 98.20 | 98.50 | 98.60 | 97.80 |
| atpF | gene | 26775 | 26919 | 145 | 98.70 | 98.60 | 98.60 | 99.30 | 98.90 |
| rps14 | gene | 36402 | 36704 | 303 | 98.70 | 98.30 | 99.70 | 99.70 | 98.70 |
| atpE | gene | 51899 | 53793 | 1895 | 98.80 | 97.60 | 99.00 | 98.60 | 96.50 |
| cemA | gene | 60901 | 61590 | 690 | 98.80 | 97.80 | 98.80 | 98.60 | 96.20 |
| psbB | gene | 72059 | 73576 | 1518 | 98.80 | 97.50 | 98.60 | 98.90 | 97.00 |
| atpB | gene | 52297 | 53793 | 1497 | 98.80 | 97.60 | 99.00 | 98.60 | 96.50 |
| psbA | gene | 490 | 1551 | 1062 | 98.90 | 99.20 | 99.40 | 99.50 | 99.00 |
| psbK | gene | 7725 | 7904 | 180 | 98.90 | 98.90 | 98.90 | 98.30 | 99.40 |
| petN | gene | 10200 | 10289 | 90 | 98.90 | 98.90 | 98.90 | 83.60 | 96.70 |
| rpoB | gene | 12837 | 16019 | 3183 | 98.90 | 97.90 | 98.90 | 99.00 | 97.50 |
| rpl33 | gene | 67268 | 67467 | 200 | 99.00 | 94.70 | 99.50 | 98.50 | 98.00 |
| rps16 | gene | 81480 | 81890 | 411 | 99.00 | 98.30 | 99.00 | 99.00 | 97.80 |
| ycf15 | gene | 99792 | 99983 | 192 | 99.00 | 97.90 | 99.00 | 99.00 | 97.40 |
| ndhI | gene | 117367 | 117867 | 501 | 99.00 | 97.80 | 99.00 | 98.60 | 97.60 |
| psbI | gene | 8314 | 8424 | 111 | 99.10 | 99.10 | 99.10 | 98.20 | 97.30 |
| atpI | gene | 24260 | 25003 | 744 | 99.10 | 98.70 | 99.60 | 99.30 | 97.80 |
| ycf4 | gene | 59519 | 60073 | 555 | 99.10 | 97.50 | 99.10 | 98.90 | 98.00 |
| psbL | gene | 63838 | 63952 | 115 | 99.10 | 100.00 | 100.00 | 100.00 | 99.10 |
| ndhA | gene | 115101 | 115652 | 552 | 99.10 | 97.40 | 99.20 | 98.60 | 97.40 |
| ndhG | gene | 118218 | 118748 | 531 | 99.10 | 97.20 | 98.70 | 99.20 | 97.60 |
| atpA | gene | 28246 | 29772 | 1527 | 99.20 | 97.90 | 99.50 | 99.10 | 98.00 |
| psbD | gene | 32347 | 34777 | 1062 | 99.20 | 98.40 | 99.10 | 99.20 | 98.40 |
| psaA | gene | 39067 | 41319 | 2253 | 99.20 | 99.40 | 99.40 | 99.40 | 98.40 |
| ndhC | gene | 49453 | 49805 | 353 | 99.20 | 97.10 | 99.70 | 99.20 | 97.70 |
| psbN | gene | 74141 | 74262 | 122 | 99.20 | 99.20 | 100.00 | 100.00 | 99.20 |
| ndhH | gene | 113918 | 115099 | 1182 | 99.20 | 98.40 | 99.50 | 99.40 | 98.20 |
| psaC | gene | 119449 | 119694 | 246 | 99.20 | 97.60 | 99.20 | 99.60 | 98.00 |
| psbC | gene | 33356 | 34777 | 1422 | 99.20 | 98.40 | 99.10 | 99.20 | 98.40 |
| rps2 | gene | 23334 | 24044 | 711 | 99.30 | 98.20 | 98.70 | 99.60 | 98.70 |
| psaB | gene | 36837 | 39041 | 2205 | 99.30 | 98.80 | 99.20 | 99.40 | 98.00 |
| rps18 | gene | 67652 | 67957 | 306 | 99.30 | 99.00 | 99.00 | 99.00 | 97.70 |
| ndhE | gene | 118959 | 119264 | 306 | 99.30 | 98.40 | 99.00 | 100.00 | 98.70 |
| rpl32 | gene | 123359 | 123509 | 151 | 99.30 | 92.30 | 98.70 | 94.70 | 97.40 |
| psbZ | gene | 35415 | 35603 | 189 | 99.50 | 100.00 | 100.00 | 100.00 | 97.90 |
| rps4 | gene | 45301 | 45906 | 606 | 99.50 | 98.00 | 99.50 | 99.20 | 98.20 |
| petB | gene | 74711 | 74716 | 648 | 99.50 | 99.30 | 99.80 | 99.50 | 99.10 |
| rpl2 | gene | 84970 | 85320 | 351 | 99.50 | 99.70 | 99.90 | 99.70 | 99.60 |
| ycf3 | gene | 42056 | 43976 | 507 | 99.50 | 98.90 | 98.70 | 99.30 | 98.40 |
| atpH | gene | 26169 | 26414 | 246 | 99.60 | 99.20 | 96.60 | 100.00 | 99.20 |
| petD | gene | 76326 | 76333 | 483 | 99.60 | 99.00 | 99.70 | 99.50 | 98.90 |
| rpl23 | gene | 86382 | 86649 | 268 | 99.60 | 99.30 | 99.60 | 99.60 | 99.30 |
| rps7 | gene | 97425 | 97892 | 468 | 99.60 | 99.10 | 99.80 | 99.60 | 99.40 |
| ndhB | gene | 138364 | 140566 | 1533 | 99.60 | 99.60 | 99.80 | 100.00 | 99.50 |
| psbM | gene | 10758 | 10862 | 105 | 100.00 | 98.10 | 98.10 | 99.00 | 98.10 |
| psbJ | gene | 63574 | 63689 | 116 | 100.00 | 99.10 | 100.00 | 100.00 | 100.00 |
| psbF | gene | 63977 | 64096 | 120 | 100.00 | 99.20 | 100.00 | 100.00 | 100.00 |
| psbE | gene | 64106 | 64357 | 252 | 100.00 | 99.60 | 99.60 | 100.00 | 99.60 |
| psaJ | gene | 66726 | 66850 | 125 | 100.00 | 97.00 | 99.30 | 100.00 | 97.00 |
| rps12-5' | gene | 69316 | 69429 | 114 | 100.00 | 100.00 | 100.00 | 100.00 | 99.10 |
| infA | gene | 80080 | 80313 | 234 | 100.00 | 98.70 | 100.00 | 99.10 | 96.20 |
| ndhD-ccsA | intergenic | 121311 | 121552 | 241 | 76.40 | 75.30 | 77.20 | 76.10 | 80.80 |
| psbI-trnS(GCU) | intergenic | 8425 | 8586 | 162 | 79.10 | 78.00 | 80.20 | 80.20 | 79.60 |
| trnH-psbA | intergenic | 75 | 489 | 415 | 80.20 | 80.00 | 82.30 | 83.10 | 85.40 |
| ndhF-ycf1 | intergenic | 126721 | 126929 | 208 | 80.30 | 79.30 | 81.80 | 77.90 | 74.50 |
| ndhI-ndhG | intergenic | 117868 | 118217 | 392 | 81.40 | 82.40 | 86.90 | 85.90 | 77.40 |
| atpA-trnR | intergenic | 29773 | 29898 | 126 | 81.70 | 92.90 | 92.10 | 81.80 | 86.00 |
| accD-psaI | intergenic | 58330 | 59019 | 745 | 82.60 | 89.30 | 88.30 | 81.30 | 81.30 |
| rps16-trnQ | intergenic | 6322 | 7269 | 1016 | 84.60 | 88.30 | 90.70 | 91.30 | 83.80 |
| trnM(CAU)-atpE | intergenic | 51691 | 51896 | 214 | 85.00 | 84.90 | 90.60 | 90.10 | 85.00 |
| trnL-rpl32 | intergenic | 122719 | 123341 | 638 | 85.60 | 84.30 | 85.50 | 89.50 | 86.30 |
| petA-psbJ | intergenic | 62795 | 63569 | 813 | 85.90 | 100.00 | 88.05 | 88.50 | 85.10 |
| trnS-trnC | intergenic | 8675 | 9328 | 654 | 86.10 | 82.70 | 85.70 | 88.10 | 82.50 |
| rpl32-ndhF | intergenic | 123510 | 123987 | 502 | 86.30 | 86.70 | 90.50 | 84.40 | 84.60 |
| cemA-petA | intergenic | 61591 | 61831 | 241 | 86.70 | 83.50 | 90.90 | 86.90 | 88.20 |
| rbcL-accD | intergenic | 56232 | 56800 | 604 | 86.90 | 88.10 | 93.20 | 89.10 | 79.50 |
| psbZ-trnG | intergenic | 35604 | 35914 | 334 | 87.10 | 89.10 | 95.30 | 93.80 | 87.00 |
| psbM-trnD | intergenic | 10946 | 11492 | 553 | 87.20 | 81.70 | 92.90 | 95.00 | 84.30 |
| ccsA-trnL | intergenic | 122522 | 122638 | 118 | 87.30 | 87.50 | 89.20 | 87.30 | 82.10 |
| trnT(UGU)-trnL(UAA) | intergenic | 46256 | 46819 | 573 | 87.40 | 84.80 | 87.00 | 88.50 | 83.80 |
| ndhC-trnV(UAC) | intergenic | 49806 | 50676 | 886 | 87.50 | 81.20 | 91.70 | 89.00 | 84.00 |
| trnW-trnP | intergenic | 66169 | 66304 | 136 | 87.50 | 92.10 | 91.80 | 93.50 | 84.40 |
| rpl22-rps19 | intergenic | 84340 | 84507 | 171 | 87.50 | 90.20 | 89.10 | 85.20 | 83.80 |
| petN-psbM | intergenic | 10290 | 10600 | 334 | 87.70 | 79.30 | 91.00 | 94.30 | 87.70 |
| psbB-psbT | intergenic | 73827 | 73955 | 142 | 88.20 | 86.30 | 86.70 | 84.20 | 94.40 |
| trnC-petN | intergenic | 9401 | 10199 | 799 | 88.50 | 81.30 | 85.20 | 83.60 | 79.40 |
| psaJ-rpl33 | intergenic | 66861 | 67267 | 429 | 88.60 | 83.60 | 90.40 | 81.90 | 84.00 |
| psaC-ndhD | intergenic | 119695 | 119807 | 114 | 88.60 | 89.70 | 90.80 | 94.80 | 93.00 |
| trnS-rps4 | intergenic | 44999 | 45300 | 314 | 88.70 | 90.30 | 95.20 | 88.60 | 88.30 |
| rpl33-rps18 | intergenic | 67469 | 67651 | 193 | 89.10 | 91.40 | 88.10 | 92.40 | 75.30 |
| trnK-rps16 | intergenic | 4385 | 5183 | 849 | 89.60 | 86.10 | 91.70 | 90.80 | 85.70 |
| rps18-rpl20 | intergenic | 67958 | 68202 | 262 | 89.70 | 94.80 | 97.20 | 90.10 | 82.80 |
| ycf4-cemA | intergenic | 60074 | 60900 | 859 | 90.20 | 86.90 | 89.30 | 88.10 | 87.80 |
| atpI-atpH | intergenic | 25004 | 26168 | 1200 | 90.30 | 87.20 | 85.50 | 92.20 | 80.00 |
| clpP-psbB | intergenic | 71617 | 72058 | 472 | 90.70 | 90.00 | 94.10 | 93.70 | 92.70 |
| ycf3-trnS | intergenic | 43977 | 44232 | 270 | 90.80 | 84.00 | 93.70 | 89.40 | 84.70 |
| trnI-psbD | intergenic | 31247 | 32346 | 1146 | 91.00 | 87.10 | 95.00 | 91.70 | 83.80 |
| rps125'-clpP | intergenic | 69430 | 69584 | 161 | 91.30 | 87.40 | 92.60 | 82.20 | 78.20 |
| rpl36-infA | intergenic | 79625 | 80072 | 448 | 91.50 | 93.80 | 91.70 | 92.10 | 88.60 |
| infA-rps8 | intergenic | 80314 | 80434 | 121 | 91.60 | 90.70 | 92.60 | 99.20 | 92.60 |
| atpB-rbcL | intergenic | 53794 | 54146 | 353 | 91.70 | 89.70 | 93.80 | 92.70 | 88.20 |
| trnF-ndhJ | intergenic | 47895 | 48121 | 236 | 91.90 | 81.80 | 86.20 | 91.10 | 78.90 |
| ndhG-ndhE | intergenic | 118749 | 118958 | 211 | 91.90 | 86.50 | 94.30 | 91.20 | 86.10 |
| trnG-trnfM | intergenic | 35991 | 36165 | 185 | 92.40 | 80.80 | 93.50 | 91.30 | 83.50 |
| trnfM-rps14 | intergenic | 36242 | 36401 | 160 | 92.50 | 92.60 | 92.00 | 92.60 | 88.30 |
| trnR-trnG | intergenic | 29971 | 30163 | 193 | 92.70 | 84.30 | 95.90 | 94.30 | 76.70 |
| psbK-psbI | intergenic | 7905 | 8313 | 418 | 92.80 | 92.70 | 95.10 | 89.40 | 86.80 |
| rpoC2-rps2 | intergenic | 23085 | 23333 | 254 | 92.90 | 85.80 | 92.10 | 88.60 | 80.70 |
| atpH-atpF | intergenic | 26415 | 26774 | 372 | 93.50 | 92.20 | 94.10 | 92.00 | 82.70 |
| psbE-petL | intergenic | 64358 | 65597 | 1259 | 94.00 | 90.30 | 95.00 | 94.70 | 87.30 |
| rps16-rps3 | intergenic | 81891 | 83073 | 1204 | 94.00 | 91.60 | 93.50 | 93.70 | 88.20 |
| petD-rpoA | intergenic | 77523 | 77925 | 414 | 94.20 | 92.30 | 94.00 | 93.00 | 91.50 |
| ycf2-trnL | intergenic | 93838 | 94018 | 181 | 94.40 | 93.10 | 96.60 | 96.10 | 92.30 |
| psbA-trnK | intergenic | 1552 | 1754 | 203 | 94.50 | 79.50 | 84.70 | 93.30 | 79.40 |
| trnL-trnF | intergenic | 47343 | 47689 | 352 | 95.20 | 88.10 | 97.70 | 88.60 | 86.70 |
| rps4-trnT | intergenic | 45907 | 46183 | 284 | 96.10 | 92.80 | 85.80 | 91.00 | 90.10 |
| psaI-ycf4 | intergenic | 59131 | 59518 | 388 | 96.10 | 88.40 | 90.80 | 90.80 | 91.60 |
| rps14-psaB | intergenic | 36705 | 36836 | 132 | 96.20 | 96.20 | 99.20 | 98.50 | 95.50 |
| ndhE-psaC | intergenic | 119338 | 119448 | 111 | 96.40 | 92.70 | 99.10 | 95.70 | 97.20 |
| trnP-psaJ | intergenic | 66466 | 66725 | 260 | 96.50 | 90.80 | 92.50 | 95.80 | 91.20 |
| trnQ-psbK | intergenic | 7342 | 7679 | 339 | 96.80 | 92.60 | 96.80 | 96.40 | 92.10 |
| rpl14-rps16 | intergenic | 81384 | 81479 | 96 | 96.90 | 88.00 | 87.80 | 88.60 | 87.20 |
| trnL-ndhB | intergenic | 94263 | 94837 | 582 | 97.10 | 92.30 | 98.30 | 98.80 | 93.80 |
| trnD-trnY | intergenic | 11571 | 11683 | 113 | 97.30 | 91.00 | 98.20 | 97.30 | 66.70 |
| rpl20-rps12 | intergenic | 68584 | 69306 | 723 | 97.60 | 93.60 | 96.60 | 97.20 | 93.60 |
| trnG-trnI | intergenic | 30961 | 31101 | 141 | 97.90 | 75.40 | 86.30 | 97.90 | 70.50 |
| ycf15-trnV | intergenic | 99994 | 100278 | 289 | 97.90 | 96.70 | 97.60 | 97.30 | 97.50 |
| psbJ-psbL | intergenic | 63690 | 63837 | 148 | 98.60 | 94.60 | 98.60 | 97.30 | 91.90 |
| rps8-rpl14 | intergenic | 80841 | 80992 | 152 | 98.70 | 94.10 | 97.30 | 98.80 | 91.60 |
| rps7-ycf15 | intergenic | 97893 | 98474 | 587 | 98.70 | 97.20 | 97.40 | 98.10 | 97.00 |
| petB-petD | intergenic | 76132 | 76325 | 194 | 99.00 | 96.70 | 96.00 | 97.90 | 91.70 |
| ndhB-rps7 | intergenic | 97137 | 97424 | 288 | 99.00 | 99.00 | 99.70 | 98.30 | 97.90 |
| rps2-atpI | intergenic | 24045 | 24259 | 215 | 99.10 | 97.70 | 97.70 | 98.10 | 94.60 |
| psbH-petB | intergenic | 74587 | 74710 | 124 | 99.20 | 98.40 | 100.00 | 100.00 | 96.80 |
| psaA-ycf3 | intergenic | 41320 | 42055 | 736 | 100.00 | 88.70 | 90.70 | 91.50 | 87.40 |
| psbN-psbH | intergenic | 74263 | 74364 | 102 | 100.00 | 98.00 | 99.00 | 100.00 | 97.10 |
| rpl23-trnI | intergenic | 86650 | 86814 | 165 | 100.00 | 100.00 | 100.00 | 100.00 | 96.40 |
| trnI-ycf2 | intergenic | 86892 | 86994 | 103 | 100.00 | 100.00 | 100.00 | 100.00 | 100.00 |
| ndhA intrion | intron | 115653 | 116748 | 1096 | 80.60 | 87.60 | 91.40 | 93.00 | 85.20 |
| rps16 intron | intron | 5468 | 6281 | 841 | 92.70 | 87.40 | 94.60 | 94.00 | 89.20 |
| petB intron | intron | 74717 | 75483 | 778 | 95.20 | 92.70 | 95.40 | 95.50 | 88.70 |
| petD intron | intron | 76334 | 77047 | 719 | 96.50 | 89.70 | 97.60 | 94.90 | 88.40 |
| atpF intron | intron | 26920 | 27625 | 713 | 97.60 | 95.70 | 98.20 | 98.60 | 94.70 |
| rpl2 intron | intron | 85597 | 85776 | 180 | 99.40 | 99.40 | 99.40 | 99.40 | 99.40 |
| ndhB intron | intron | 95690 | 96359 | 670 | 99.90 | 99.60 | 99.10 | 100.00 | 99.70 |
