# Supplementary material for: Complete Chloroplast Genome Sequence of a Major Invasive Species, Crofton Weed (Ageratina adenophora)
Source: PLoS One. 2012 May 11;7(5):e36869. doi: 10.1371/journal.pone.0036869 (PMC3350484; doi:10.1371/journal.pone.0036869)
Supplement: Table S3 — Repeat sequences in the A . adenophora chloroplast genomes. (DOC) [file pone.0036869.s003.doc]

**Table S3.** Repeat sequences in the *A.adenophora* chloroplast genomes.

| **Length (bp)** | **Repeat Type** | **Direct** | **Position A** | **Position B** | **Location** | **Genomes region** |
| --- | --- | --- | --- | --- | --- | --- |
| 16 | tandem(2×^*^) | F | 126 | 138 | *trnH-psbA* | intergenic spacer |
| 16 | tandem(2×) | F | 364 | 381 | *trnH-psbA* | intergenic spacer |
| 29 | tandem(polyT) | F | 1980 | 2008 | *trnK(UUU)* | intron |
| 15 | tandem(2×) | F | 2012 | 2027 | *trnK(UUU)* | intron |
| 24 | tandem(2×) | F | 25331 | 25355 | *atpI-atpH* | intergenic spacer |
| 22 | tandem(2×) | F | 30123 | 30145 | *trnR(UCU)-trnG(UCC)* | intergenic spacer |
| 22 | tandem(2×) | F | 30335 | 30357 | *trnG(UCC)* | intron |
| 17 | tandem(2×) | F | 34994 | 35011 | *psbC-trnS(GCU))* | intergenic spacer |
| 28 | tandem(polyA) | F | 41798 | 41825 | *psaA-ycf3* | intergenic spacer |
| 22 | tandem(2×) | F | 56015 | 56037 | *ndhC-trnV(UAC)* | intergenic spacer |
| 22 | tandem（polyT） | F | 12760 | 12782 | *trnE(UUC)-rpoB* | intergenic spacer |
| 22 | tandem（polyT） | F | 28201 | 28223 | *atpF-atpA* | intergenic spacer |
| 15 | tandem(2×) | F | 54150 | 54165 | *atpB-rbcL* | intergenic spacer |
| 20 | tandem(2×) | F | 54162- | 54182 | atpB-rbcL | intergenic spacer |
| 15 | tandem(2×) | F | 54406 | 54421 | atpB-rbcL | intergenic spacer |
| 18 | tandem(2×) | F | 56647- | 56665 | rbcL-accD | intergenic spacer |
| 15 | Tandem(6×) | F | 57319 | 57334 | accD | cds |
| 20 | tandem(2×) | F | 58918 | 58938 | accD-psaI | intergenic spacer |
| 15 | tandem(2×) | F | 61719 | 61734 | cemA-petA | intergenic spacer |
| 21 | tandem(2×) | F | 67657 | 67678 | rps18 | cds |
| 19 | tandem(2×) | F | 74094- | 74113 | psbT-psbN | intergenic spacer |
| 16 | tandem(2×) | F | 84602- | 84618 | rpl22-rps19 | intergenic spacer |
| 85 | tandem(2×) | F | 86283 | 86368 | rp12-rpl23 | intergenic spacer |
| 18 | tandem(3×) | F | 91947- | 91965 | ycf2 | cds |
| 32 | tandem(2×) | F | 106940 | 106972 | 4.5-5S rRNA | intergenic spacer |
| 24 | tandem(2×) | F | 108583- | 108607 | ycf1 | cds |
| 21 | tandem(2×) | F | 112089 | 112110 | ycf1 | cds |
| 19 | tandem(2×) | F | 116355 | 116374 | ndhA | cds |
| 15 | tandem(2×) | F | 122826 | 122841 | trnL-rpl32 | intergenic spacer |
| 16 | tandem(2×) | F | 123084 | 123099 | trnL-rpl32 | intergenic spacer |
| 18 | tandem(3×) | F | 126787 | 126805 | ndhF-ycf1 | intergenic spacer |
| 219 | dispersed | P | 79620 | 80620 | *rps136-inf;inf-rps8* | intergenic spacer |
| 145 | dispersed | F | 26774 | 27625 | atpF | cds |
| 144 | dispersed | F | 84339 | 84700 | rpl22-rps19; rps19 | intergenic spacer |
| 101 | dispersed | P | 85595 | 85859 | rpl2 | cds |
| 68 | dispersed | P | 11331 | 12512 | psbM-trnD(GUC);trnE(UUC)-rpoB | intergenic spacer |
| 63 | dispersed | P | 94029 | 94180 | *ycf2-*trnL(CAA) | intergenic spacer |
| 60 | dispersed | F | 8583 | 35011 | trnS(GCU); psbC-Z | intergenic spacer |
| 60 | dispersed | P | 10936 | 11010 | psbM-trnD(GUC) | intergenic spacer |
| 58 | dispersed | P | 71286 | 73775 | clp intron; psbB-psbT | intron |
| 57 | dispersed | F | 47689 | 47823 | trnF(GAA) | intron |
| 50 | dispersed | F | 47639 | 50675 | TrnF(GAA); ndhC-trnV(UAC) | intergenic spacer |
| 49 | dispersed | F | 8625 | 44936 | trnS(GCU);trnS(GAA)-rps4 | intergenic spacer |
| 48 | dispersed | P | 11758 | 30875 | trnY(GUA);trnG(UCC) | intergenic spacer |
| 51 | dispersed | F | 81061 | 84259 | rpl14;rpl22-rps19 | intergenic spacer |
| 45 | dispersed | F | 5415 | 6280 | rps16 | intron |
| 44 | dispersed | P | 57318 | 57363 | accD | cds |
| 44 | dispersed | F | 81014 | 84212 | rps14; rpl22-rps19 | intergenic spacer |
| 41 | dispersed | P | 18467 | 18802 | rpoC1 | intron |
| 43 | dispersed | P | 54464 | 58615 | atpB-rbcL; accD-psaI | intergenic spacer |
| 40 | dispersed | P | 83335 | 98727 | rps3; rps7-ycf15 | cds |
| 46 | dispersed | P | 10617 | 10617 | petN-psbM | intergenic spacer |
| 39 | dispersed | P | 43194 | 83336 | ycf3 intron; rps3 | intron |
| 39 | dispersed | F | 43194 | 98727 | ycf3 intron;rps7-ycf15 | intron |
| 35 | dispersed | F | 43190 | 119300 | ycf3 intron; rps7-ycf15 | intron |
| 32 | dispersed | F | 9296 | 9449 | trnS(GCU)-trnC(GCA);  trnC(GCA)-petN | intergenic spacer |
| 31 | dispersed | F | 77717 | 77958 | petD-rpoA; rpoA | cds |
| 31 | dispersed | F | 98727 | 119304 | rps7-ycf15; ndhE-psaC | intergenic spacer |
| 31 | dispersed | P | 119304 | 136741 | ndhE-psaC; ycf15-rps7 | intergenic spacer |

*copy number

NOTE: ‘Direct’ means the direction of the repeat units. ‘F’ is forward, and ‘P’ is palindromic. 'Position A' and 'Position B means the repeat I start site and repeat II start site, respectively. And the values within the columns mean the sequence site in the genome.
