# Supplementary material for: Complete Chloroplast Genome Sequence of a Major Invasive Species, Crofton Weed (Ageratina adenophora)
Source: PLoS One. 2012 May 11;7(5):e36869. doi: 10.1371/journal.pone.0036869 (PMC3350484; doi:10.1371/journal.pone.0036869)
Supplement: Table S4 — The GenBank accession numbers of all the 33 cp genomes used for phylogenetic analysis. (DOC) [file pone.0036869.s004.doc]

Table S4. The GenBank accession numbers of all the 33 cp genomes used for phylogenetic analysis.

| No | GenBank Accession number | Species name |
| --- | --- | --- |
| 1 | NC_011828 | *Trifolium subterraneum* |
| 2 | NC_003119 | *Medicago truncatula* |
| 3 | NC_011163 | *Cicer arietinum* |
| 4 | NC_002694 | *Lotus japonicus* |
| 5 | NC_009259 | *Phaseolus vulgaris* |
| 6 | NC_007144 | *Cucumis sativus* |
| 7 | NC_008334 | *Citrus sinensis* |
| 8 | NC_000932 | *Arabidopsis thaliana* |
| 9 | NC_008641 | *Gossypium barbadense* |
| 10 | NC_007944 | *Gossypium hirsutum* |
| 11 | NC_008115 | *Eucalyptus globulus* |
| 12 | NC_010358 | *Oenothera elata subsp. Hookeri* |
| 13 | NC_001879 | *Nicotiana tabacum* |
| 14 | NC_004561 | *Atropa belladonna* |
| 15 | NC_007943 | *Solanum bulbocastanum* |
| 16 | NC_007898 | *Solanum lycopersicum* |
| 17 | NC_008325 | *Daucus carota* |
| 18 | NC_006290 | *Panax ginseng* |
| 19 | NC_007578 | *Lactuca sativa* |
| 20 | NC_015543 | *Jacobaea vulgaris* |
| 21 | NC_015621 | *Ageratina adenophora* |
| 22 | NC_010601 | *Guizotia abyssinica* |
| 23 | NC_007977 | *Helianthus annuus* |
| 24 | NC_013553 | *Parthenium argentatum* |
| 25 | NC_002202 | *Spinacia oleracea* |
| 26 | NC_008796 | *Ranunculus macranthus* |
| 27 | NC_007407 | *Acorus calamus* |
| 28 | NC_002762 | *Triticum aestivum* |
| 29 | NC_008155 | *Oryza sativa* |
| 30 | NC_006084 | *Saccharum officinarum* |
| 31 | NC_001666 | *Zea mays* |
| 32 | NC_008788 | *Nuphar advena* |
| 33 | NC_006050 | *Nymphaea alba* |
